# Predictive Power of Baseline [^18^F]FDG PET/CT for Adverse Events in DLBCL Patients Undergoing CAR-T Cell Therapy

**DOI:** 10.3390/diagnostics15162025

**Published:** 2025-08-13

**Authors:** Helena A. Peters, Emil Novruzov, Ben-Niklas Bärmann, Daniel Weiss, Matthias Boschheidgen, Vivien Lorena Ivan, Nora Liebers, Johannes Fischer, Eduards Mamlins, Aleksandar Radujkovic, Guido Kobbe, Julian Kirchner, Peter Minko, Kathrin Nachtkamp, Paul Jäger, Christina Antke, Frederik L. Giesel, Sascha Dietrich, Gerald Antoch, Kai Jannusch

**Affiliations:** 1 Department of Diagnostic and Interventional Radiology, Medical Faculty, University Hospital Düsseldorf, Heinrich-Heine-University Düsseldorf, D-40225 Düsseldorf, Germany; emil.novruzov@med.uni-duesseldorf.de (E.N.); danielarvid.weiss@med.uni-duesseldorf.de (D.W.); matthias.boschheidgen@med.uni-duesseldorf.de (M.B.); vivienlorena.ivan@med.uni-duesseldorf.de (V.L.I.); eduards.mamlins@med.uni-duesseldorf.de (E.M.); julian.kirchner@med.uni-duesseldorf.de (J.K.); peter.minko@med.uni-duesseldorf.de (P.M.); christina.antke@med.uni-duesseldorf.de (C.A.); frederik.giesel@med.uni-duesseldorf.de (F.L.G.); antoch@med.uni-duesseldorf.de (G.A.); kai.jannusch@med.uni-duesseldorf.de (K.J.); 2 Department of Nuclear Medicine, Medical Faculty, University Hospital Düsseldorf, Heinrich-Heine-University Düsseldorf, D-40225 Düsseldorf, Germany; 3 Department of Hematology, Oncology and Clinical Immunology, Medical Faculty, University Hospital Düsseldorf, Heinrich-Heine-University Düsseldorf, D-40225 Düsseldorf, Germany; ben-niklas.baermann@med.uni-duesseldorf.de (B.-N.B.); nora.liebers@med.uni-duesseldorf.de (N.L.); itz-sekretariat@med.uni-duesseldorf.de (J.F.); aleksandar.radujkovic@med.uni-duesseldorf.de (A.R.); kobbe@med.uni-duesseldorf.de (G.K.); kathrin.nachtkamp@med.uni-duesseldorf.de (K.N.); paulsebastian.jaeger@med.uni-duesseldorf.de (P.J.); sascha.dietrich@med.uni-duesseldorf.de (S.D.); 4 Center for Integrated Oncology Aachen Bonn Cologne Düsseldorf (CIO ABCD), D-53127 Bonn, Germany

**Keywords:** CAR-T, FDG-PET/CT, SUVmax, cytokine release syndrome, immune effector cell-associated neurotoxicity syndrome

## Abstract

**Objectives**: Evaluation of the predictive potential of pre-CAR-T [^18^F]FDG PET/CT in Diffuse Large B-Cell Lymphoma (DLBCL) patients concerning Cytokine Release Syndrome (CRS) and Immune Effector Cell-associated Neurotoxicity Syndrome (ICANS). **Methods**: Eighteen DLBCL patients (mean age: 60 ± 12 years) who underwent pre-therapeutic [^18^F]FDG-PET/CT and CAR-T cell therapy were retrospectively included. Median follow-up time was ten months (IQR6-16) after CAR-T cell infusion. Age, sex, serum lactate dehydrogenase (LDH), interleukin-6 (IL-6), C-reactive protein (CRP), and modified Endothelial Activation and Stress Index (mEASIX) were obtained. Potential occurrence of CRS/ICANS and the SUV_max_ were evaluated. Pearson and Spearman correlations, group comparisons (Mann–Whitney U-test) and the odds ratio (OR) were calculated. P values below 0.05 were defined as statistically significant and 95%-confidence intervals (CI) were calculated. **Results**: Pre-therapeutic SUV_max_ correlated positively with LDH (r = 0.5; *p* = 0.02), with the grade of CRS (r = 0.5; *p* = 0.03) and with the grade of ICANS (r = 0.6; *p* = 0.01). Appearance of ICANS was significantly correlated with pre-therapeutic SUV_max_ (*p* = 0.03; U = 7.0; Z = −2.2). Using ROC analysis and Youden’s index, an SUV_max_ threshold of 17 (AUC: 0.865; *p* < 0.01) was defined. Patients exceeding a pre-therapeutic SUV_max_ of 17 had a significantly higher risk of CRS grade > 1 (OR = 22; CI 2, 314; *p* = 0.03) and ICANS grade > 1 (OR = 18; CI 1, 271; *p* = 0.04). **Conclusions**: Pre-therapeutic SUV_max_ may be a useful marker for identifying DLBCL patients at risk for CRS and ICANS.

## 1. Introduction

The clinical implementation of CD19-targeting Chimeric Antigen Receptor (CAR)-T cell therapy is promising for patients with relapsed/refractory Diffuse Large B-Cell Lymphoma (r/r) DLBCL [1]. Despite high remission rates (50–65%) after CAR-T cell therapy in DLBCL patients, improved understanding and risk stratification of therapy-associated adverse events such as cytokine release syndrome (CRS) and immune effector cell-associated neurotoxicity syndrome (ICANS), especially with regard to potentially life-threatening symptoms, are essential [2,3,4,5]. Prognostic predictors are required to identify DLBCL patients at risk for CAR-T cell therapy-associated adverse events, in order to optimize patient-centered therapeutic management and minimize treatment-related morbidity [6,7].

CRS is a systemic inflammatory reaction to CAR-T cell therapy, occurring in approximately 58–93%, of patients, most often within the first week after infusion. Symptoms range from fever and hypotension to hypoxia and multi-organ failure [7,8,9]. In contrast, ICANS manifests as neurological symptoms, including confusion, tremor, aphasia, and coma, in 21–66% of patients. Both are frequently associated with high inflammatory cytokine levels [8,9,10,11].

According to guidelines 2-[^18^F]Fluoro-2-Deoxy-D-Glucose Positron Emission Tomography/Computed Tomography ([^18^F]FDG-PET/CT) is currently the imaging modality of choice for r/r DLBCL patients, used for baseline and follow-up evaluations to assess treatment response and prognosis [12,13]. [^18^F]FDG-PET/CT offers insights into metabolism of healthy immune system structures, which are known to be involved in CAR-T cell-induced adverse events [14]. FDG uptake reflects increased glucose metabolism not only in malignant tissues, but also in sites of active inflammation, due to the upregulation of glucose transporters and glycolytic enzymes in both proliferating tumor cells and activated immune cells [15,16]. This shared metabolic pathway is particularly relevant for differentiating therapy-induced inflammatory toxicity from residual or recurrent malignancy in patients after CAR-T cell therapy.

While clinical scores such as the modified Endothelial Activation and Stress Index (mEASIX) are available to estimate the risk of toxicity after CAR-T cell therapy, these biomarkers primarily reflect systemic inflammation and endothelial stress [17,18]. Imaging-based parameters, such as SUV_max_ from [^18^F]FDG-PET/CT may offer additional clinical value by providing complementary, morphology-related information prior to treatment initiation.

Consequently, this study aims to evaluate whether pre-therapeutic SUV_max_, derived from routine [^18^F]FDG-PET/CT imaging, can serve as an early, clinically applicable biomarker to stratify patients at risk of CAR-T cell therapy-associated adverse events, particularly CRS and ICANS, prior to treatment initiation and decision-making.

## 2. Materials and Methods

### 2.1. Patients

The study was approved by the institutional review board of the University of Duesseldorf (study number: 2023-2618) and it was performed in accordance with the Declaration of Helsinki [19].

Retrospective data of 18 [^18^F]FDG-PET/CT scans (18 patients) were acquired before CAR-T cell infusion (18 (IQR 9–48) days before CAR-T cell infusion and 14 (IQR 10–41) days before lymphodepletion), between June 2020 and September 2023. Written informed consent was waived in accordance with the institutional review board because of the retrospective study design. Inclusion criteria were defined as follows: (i) age above 18 years, (ii) CD19-targeted CAR-T cell therapy for relapsed or refractory DLBCL, (iii) recorded patient characteristics as outlined in the section “Patients’ Demographics/Characteristics, Follow-up and Clinical Data”. Exclusion criteria include (i) primary DLBCL, and (ii) further malignancies.

**Figure 1:**
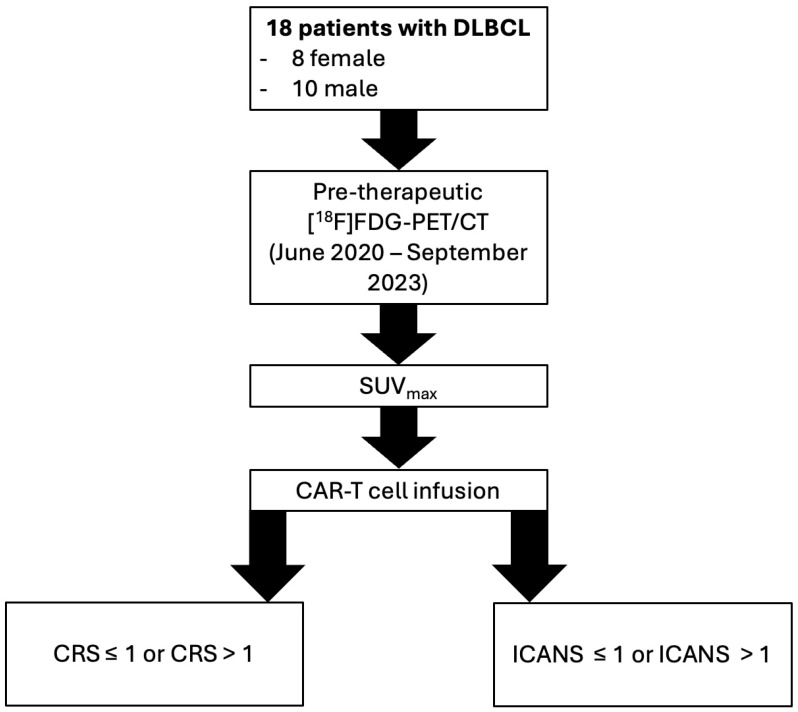
Workflow diagram illustrating the study design, including patient selection, PET/CT imaging, and correlation of SUV_max_ with CRS and ICANS. Notes: DLBCL: Diffuse Large B-Cell Lymphoma; [^18^F]FDG-PET/CT: 2-[^18^F]Fluoro-2-Deoxy-D-Glucose Positron Emission Tomography/Computed Tomography; CAR-T: CD19-targeting Chimeric Antigen Receptor (CAR)-T cell; CRS: Cytokine Release Syndrome; ICANS: Immune Effector Cell-associated Neurotoxicity Syndrome.

To visualize the study process, a workflow diagram is provided in Figure 1, detailing patient selection, PET/CT evaluation, and correlation with clinical outcomes.

### 2.2. PET/CT Imaging

All [^18^F]FDG-PET/CT data were acquired on a Biograph mCT 128 (Siemens Healthineers, Erlangen, Germany). The average delay was 60 ± 5.17 min after injection of a bodyweight-adapted dosage of [^18^F]FDG (3 MBq/kg bodyweight). To ensure blood glucose levels below 150 mg/dL, blood samples were obtained, and patients needed to fast for six hours prior to injection of [^18^F]-FDG. The mean activity applied to patients at [^18^F]FDG-PET/CT was 230  ±  38 MBq. PET/CT was performed with a whole-body scan. Weight-adapted iodinated contrast medium (Accupaque 300, GE Healthcare, Chicago, IL, USA) was used in 9/18 (44%) [^18^F]FDG-PET/CT scans in those patients without prior diagnostic (contrast-enhanced) whole-body CT. CT acquisition started 70 s after intravenous injection of the contrast agent. Automated tube current modulation was activated in all scans (presets 120 kV, 190 reference mAs, collimation 128 × 0.6 mm, pitch 0.8, slice thickness 1.5 mm). An additional diagnostic low-dose lung tissue scan in deep inspiration was added to all [^18^F]FDG-PET/CTs to improve pulmonary imaging [20]. PET data were acquired for 3 min in supine position (matrix size 200 × 200, axial field of view 21.8 cm and a Gaussian filter of 2.0 mm). Attenuation correction was performed and iterative reconstruction using ordered subset expectation maximization was used with the following presets: 4 iterations and 8 subsets.

### 2.3. Image Analysis

A board-certified radiologist experienced in nuclear medicine diagnostics and a board-certified nuclear medicine physician did further data evaluation of the acquired [^18^F]FDG-PET/CT datasets using a dedicated PACS-Workstation (IDS7; Sectra, Linköping, Sweden). The readers examined all datasets for suspicious lesions (lymphonodal/extranodal) indicative of lymphoma manifestations. Manifestations were assessed on [^18^F]PET based on visually elevated focal FDG-uptake compared to background, mediastinum and liver activity [21].

**Figure 2:**
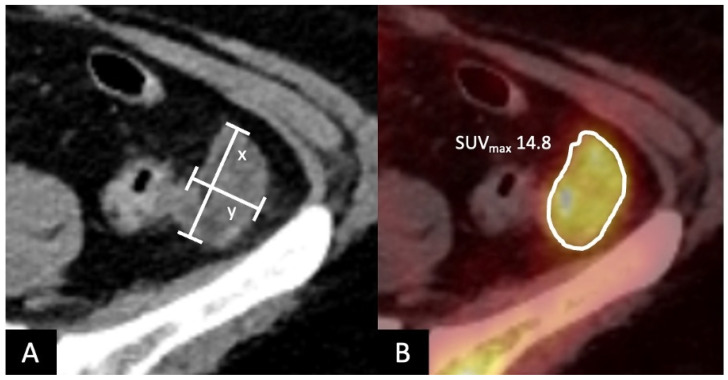
A fifty-two-year-old patient with diffuse large B-cell lymphoma (DLBCL) and abdominal lymphoma manifestation left-sided in the paracolic channel, lateral to the descending colon. Example of image analysis with (**A**) measurement of maximal- (x) and minimal- (y) diameter in mm to determine tumor volume and (**B**) measurement of the SUV_max_ in the tumor area with the highest metabolic activity using a volume of interest (VOI).

The [^18^F]FDG-PET/CT data for each patient were analyzed to determine (i) the maximum and minimum diameter of maximum six lymphoma manifestations, (ii) tumor volume/SPD (sum of the product of diameters) in mm^2^ and (iii) the SUV_max_ of the lesion with the highest metabolic activity using a manually placing area-adapted volume of interest (VOI) around the visually identified lesion. Measurement of the SUV_mean_ was omitted, following the approach of previous studies [22,23]. An example of data acquisition is illustrated in Figure 2. Additionally, the SUV_max_ of liver and spleen parenchyma and SUV_max_ of the mediastinal blood pool were measured at pre-defined areas, each with a predefined VOI.

### 2.4. Patients’ Demographics/Characteristics, Follow-Up and Clinical Data

Patient demographics, clinical data (Lactate dehydrogenase [LDH], interleukin 6 [IL-6], C-reactive protein (CRP), modified Endothelial Activation and Stress Index (mEASIX), Ann Arbor stage [AAS]), start-/endpoint of therapy and side effects [CRS and ICANS] were obtained from each patient.

The grades of the CRS and ICANS were evaluated for each patient. CRS and ICANS are categorized according to the Consensus Guidelines of the American Society of Transplantation and Cellular Therapy (see Table 1) [24]. Additionally, the serum LDH in U/L, IL-6 in ng/L, CRP in mg/dL and the mEASIX and were determined for each patient.

### 2.5. Statistical Analysis

SPSS Statistics 26 (IBM Inc., Armonk, NY, USA) was used for statistical analyses. Descriptive statistics were obtained, with data presented as mean ± SD for normally distributed continuous variables. For non-normally distributed continuous variables, median values were reported using interquartile range (IQR, 1st quarter–3rd quarter). Pearson and Spearman correlations and group comparisons using the Mann–Whitney U-test between [^18^F]FDG-PET/CT imaging parameters towards clinical parameters (LDH, IL-6, CRP; mEASIX, CRS and ICANS) were calculated. Furthermore, the odds ratio (OR) was used to calculate the extent to which SUV_max_ had a significant impact on the occurrence of CRS and ICANS. *p*-values < 0.05 were set as statistically significant. For the odds ratio, the corresponding 95% confidence intervals (CIs) were calculated.

A priori power analyses were performed using G*Power (version 3.1) to estimate the required sample size for detecting a moderate diagnostic effect of SUV_max_ (assumed AUC = 0.80) in predicting the occurrence of CRS > 1 and ICANS > 1. The AUC was converted into an effect size (Cohen’s d = 0.85) based on established approximations. For CRS > 1, with a group ratio of 6 patients with CRS > 1 and 12 patients with CRS ≤ 1 (allocation ratio = 2.0), a total sample size of approximately 39 patients (13 in CRS > 1 and 26 in CRS ≤ 1) would be required to achieve 80% power at a significance level of α = 0.05. For ICANS > 1, with only 4 affected patients and 14 with ICANS ≤ 1 (allocation ratio = 3.5), the required total sample size increases to approximately 52 patients (13 with ICANS > 1 and 39 with ICANS ≤ 1).

Given that the current cohort consists of only 18 patients, the statistical power is limited, and the results should therefore be interpreted as exploratory and hypothesis-generating.

## 3. Results

### 3.1. Patient Characteristic and PET/CT Metabolic Parameters

Eighteen patients were included in the study. All 18 patients suffered from r/r DLBCL. The median follow-up time was ten months (IQR 6–16) after CAR-T cell infusion. Six patients had a CRS > 1 and 12 patients had a CRS ≤ 1. Four patients revealed an ICANS score above > 1, whereas 14 patients revealed an ICANS score ≤ 1. A detailed overview of patients’ demographics/characteristics and specific CAR-T cell therapy is given in Table 2.

### 3.2. SUV_max_ and Clinical Parameters

The median pre-therapeutic SUV_max_ was 9.0 (IQR 5.4–16.0; range: 1.8–37.0) and the median serum LDH was 238 U/L (IQR 18–269). At baseline the median IL-6 was 27 ng/L (IQR 8–41), CRP was 2.2 ± 3.2 mg/dL and the modified EASIX score (baseline serum LDH (U/L) × baseline CRP (mg/dL)/baseline platelets (10^9^/L)) was 1.8 (IQR 0.7–4.4).

The mean serum glucose level at the time of [^18^F]FDG-PET/CT was 105 ± 21 mg/dL. No significant correlation was observed between serum glucose levels and FDG uptake in our study population (r = −0.24; CI: −0.64, 0.26; *p* = 0.35).

No significant difference in group comparison using the Mann–Whitney U-test between the pre-therapeutic SUV_max_ of patients with a CRS of ≤1 and patients with a CRS > 1 was found. Patients with an ICANS ≤ 1 differed significantly from patients with an ICANS > 1 according to pre-therapeutic SUV_max_. An overview of group comparisons (SUV_max_) is given in Table 3.

### 3.3. Prediction of Toxicity

There was a moderate positive correlation of baseline SUV_max_ with the severity of CRS, ICANS and the level of LDH. No significant correlation was observed between baseline SUV_max_ and IL-6 levels, CRP, or the modified EASIX score. (see Table 4). Furthermore, there was no significant correlation between the pre-therapeutic SUV_max_ of the spleen and liver and the grade of CRS or ICANS (see Table 5).

**Figure 3:**
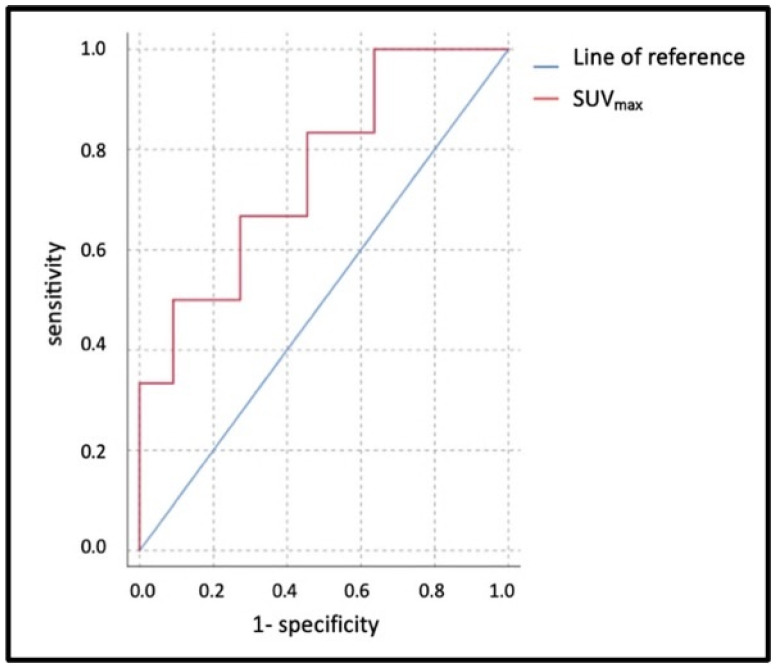
ROC analysis of pre-therapeutic SUV_max_.

ROC analysis and Youden’s Index were used to calculate 17 as the statistically significant SUV_max_ threshold value for patients at risk of a CRS > 1 or ICANS > 1 (AUC: 0.865; *p* = 0.001; sensitivity: 75%, and specificity: 93%, see Figure 3). Patients exceeding an SUV_max_ of 17 had an increased risk of CRS > 1 and ICANS > 1 (see Table 4).

## 4. Discussion

Baseline SUV_max_ from [^18^F]FDG PET/CT may be a reliable indicator to define patients at risk of developing CRS and/or ICANS in CAR-T cell therapy. An SUV_max_ threshold of more than 17 at pre-CAR-T cell therapy [^18^F]FDG PET/CT seems to predict CRS > 1 and ICANS > 1.

Data regarding potential prognostic imaging markers for assessing CRS and ICANS as the most common CAR-T cell therapy-associated adverse events are currently limited [25]. However, implementing such a promising therapy makes it crucial to identify those patients at risk of relevant side effects prior to CAR-T cell therapy. In particular, the high risk of intensive care management or death following the development of CRS or ICANS underscores the need to identify easily accessible prognostic imaging markers [26]. For that purpose, this study evaluates the potential of pre-CAR-T SUV_max_ measurements to identify patients at risk of CAR-T cell therapy toxicity (CRS or ICANS) and to increase the understanding of SUV_max_ regarding CAR-T cell therapy.

According to our data, two clinically relevant observations can be derived from the results: First, pre-therapeutic SUV_max_ is positively associated with the severity of both CRS and ICANS. Second, a pre-therapeutic SUV_max_ exceeding 17 may indicate an increased risk of developing CRS > 1 and ICANS > 1. Importantly, we found no significant correlation between serum glucose levels and SUV_max_, suggesting that FDG uptake in our cohort was not substantially influenced by variations in glycaemia within the observed range.

The relationship between prognostic metabolic imaging parameters and CAR-T cell therapy-associated side effects (CRS/ICANS) has already been investigated in individual studies with the aim of identifying patients at risk at an early stage of treatment and adjusting treatment protocols as early as possible [3,25,27,28]. Although there is one study by Zhou et al. (2022) that did not find a positive correlation between the pre-CAR-T cell therapy PET parameters and the occurrence of CAR-T therapy-related toxicity [29], several other publications are in line with our results, indicating a positive correlation between the severity of CRS and ICANS and the pre-therapeutic SUV_max_. Wang et al. (2019) observed that a higher baseline disease burden is associated with a more severe CRS [27], supported by Ababneh et al. (2024) who confirmed that a high SUV_max_ was associated with grade 3 to 4 neurological events [30]. Derlin et al. (2021) and Hong et al. (2021) were able to support these findings, as well, by presenting a correlation between higher baseline metabolism (SUV_max_) and neurotoxicity [3,25]. Additionally, in a recent analysis by Gui et al. (2024), a strong correlation was found between pre-infusion SUV_max_ and the severity of CRS [31]. This clearly supports and further validates our findings, demonstrating that baseline PET metrics, especially SUV_max_, are useful predictors for adverse events associated with CAR-T cell therapy.

The occurrence of CRS and ICANS is associated with the release of cytokines by CAR-T cells, tumor cells, mononuclear/macrophage cells, and endothelial cells. Thus, their development is marked by elevated levels of several cytokines, including interferon gamma (IFN-γ), interleukin-2 (IL-2), interleukin-6 (IL-6) and lactate dehydrogenase (LDH). These elevated cytokine levels drive the inflammatory response that leads to the onset of CRS and ICANS after CAR-T cell infusion [32,33]. Due to expected higher metabolic activity, a positive correlation between pre-therapeutic SUV_max_ and IL-6 was expected in this study. However, no significant association was found. Nonetheless, there was a positive correlation between the pre-therapeutic SUV_max_ and the baseline LDH level, indicating that the SUV_max_ and higher LDH levels may serve as indicators of the respective occurrence of CRS and ICANS [7,34]. Especially in patients with a SUV_max_ exceeding 17, stricter clinical monitoring could be helpful for early diagnosis of a potentially unfavorable therapeutic outcome.

In the context of metabolic activity, the relationship between the SUV_max_ of the spleen and liver parenchyma and the occurrence of CRS and ICANS was investigated as well. The spleen plays a crucial role in generating and nurturing immune cells, while the liver facilitates the maturation of various lymphocytes and is increasingly recognized as part of the lymphoid organ network [14,35]. However, there was no significant correlation between the SUV_max_ of the liver or spleen at baseline and the severity of CRS/ICANS, contrary to the results of Marchal et al. (2024) [14]. They demonstrated that the mean liver and spleen uptake were associated with CRS and ICANS of clinical grades 2 to 4. These differences may be explained by the smaller patient population in our study.

Considering these findings, the inclusion of pre-therapeutic SUV_max_ seems to be a helpful parameter for risk stratification of patients undergoing CAR-T cell therapy. Given that both CRS and ICANS can manifest with mild to life-threatening symptoms, the early identification of patients at risk will have a decisive impact on the decision on whether a patient is suitable for CAR-T cell therapy [36].

Our study has several limitations that should be acknowledged. First, the retrospective, single-center design introduces inherent risks of bias and unmeasured confounding. Second, the relatively small sample size limited the study’s statistical power and precluded external validation. Notably, the SUV_max_ cut-off value of ≥17 was derived and tested within the same dataset, which carries a risk of optimism bias and may overestimate its diagnostic accuracy. Furthermore, SUV_max_ was determined based on manually defined, visually adapted VOIs without application of a standardized threshold method. While this reflects routine clinical practice, the lack of a fixed delineation strategy may introduce inter-observer variability and limit reproducibility. In addition, the study was conducted at a tertiary referral center and included only patients with r/r DLBCL treated with CAR-T cell therapy, which may limit the generalizability of the results to broader, more heterogeneous clinical populations. Selection bias and spectrum limitations cannot be excluded. Future prospective multicenter studies with larger and more diverse patient cohorts are essential to validate the clinical applicability of SUV_max_-based risk stratification and to improve our understanding of CAR-T-related toxicities.

## 5. Conclusions

Pre-therapeutic SUV_max_ may serve as a useful imaging marker to identify DLBCL patients at increased risk of CRS > 1 and ICANS > 1. It may be reasonable to incorporate SUV_max_ into the clinical risk stratification process of DLBCL patients undergoing CAR-T cell therapy.

## Figures and Tables

**Table 1:** Consensus grading for cytokine release syndrome (CRS) according to the American Society for Transplantation and Cellular Therapy (ASTCT) and consensus grading for immune effector cell-associated neurotoxicity syndrome (ICANS) according to the American Society for Transplantation and Cellular Therapy (ASTCT).

Grade CRS	1	2	3	4
Temperature	≥38 °C	≥38 °C	≥38 °C	≥38 °C
Degree of hypotension	Awakens spontaneously	Awakens to voice	Awakens only to tactile stimulations	Stupor or coma
Degree of hypoxia	None	Hypotension not requiring vasopressors	Hypotension requiring one vasopressor	Hypotension requiring multiple vasopressors
Motor findings	None	Hypoxia requiring low-dose O^2^ supplementation	Hypoxia requiring high-dose O^2^ supplementation	Hypoxia requiring positive-pressure O^2^ supplementation
**Grade ICANS**	**1**	**2**	**3**	**4**
ICE score	7–9	3–6	0–2	0
Depressed level of consciousness	Awakens spontaneously	Awakens to voice	Awakens only to tactile stimulations	Stupor or coma
Seizure	N/A	N/A	Any clinical seizure focal or generalized that resolves rapidly or nonconvulsive seizures on EEG that resolves with intervention	Life-threatening prolonged seizure (<5 min) or repetitive clinical or electrical seizures without return to baseline in between
Motor findings	N/A	N/A	Life-threatening prolonged seizure (<5 min) or repetitive clinical or electrical seizures without return to baseline in between	Deep focal motor weakness such as hemiparesis or paraparesis
Elevated ICP/cerebral edema	N/A	N/A	Focal/local edema on neuroimaging	Diffuse cerebral edema on neuroimaging; decerebrate or decorticate posturing; cranial nerve VI palsy; papilledema; or Cushing’s triad

Notes: N/A indicates not applicable; ICE: immune effector cell-associated encephalopathy; ICP: intracranial pressure.

**Table 2:** Overview of patients’ demographics/characteristics, used CAR-T cell therapy and adverse events.

Patients’ Demographics/Characteristics/Side Effects	Value	Percentage (%)
Number of Patients	*n* = 18	100
Age in years		
Mean ± SD	60 ± 12	
Gender		
Female	*n* = 8/18	44
Male	*n* = 10/18	56
Initial Ann Arbor stage (AAS)		
I	*n* = 2/18	11.1
II	*n* = 1/18	5.6
III	*n* = 5/18	27.8
IV	*n* = 10/18	55.5
Cytokine Release Syndrome (CRS)		
no CRS	*n* = 2/18	11.1
1	*n* = 10/18	55.5
2	*n* = 4/18	22.2
3	*n* = 1/18	5.6
4	*n* = 1/18	5.6
Immune Effector Cell-associated Neurotoxicity Syndrome (ICANS)		
no ICANS	*n* = 14/18	77.7
1	*n* = 0/18	0
2	*n* = 2/18	11.1
3	*n* = 1/18	5.6
4	*n* = 1/18	5.6

**Table 3:** Group comparison of pre-therapeutic SUV_max_ between patients with CRS ≤ 1 and >1/ICANS ≤ 1 and >1.

	CRS ≤ 1 vs. CRS > 1	ICANS ≤ 1 vs. ICANS > 1
Mann–Whitney U	16.0	7.0
Wilcoxon W	94.0	112.0
Z	−1.9	−2.2
*p*-value	0.07	0.03 *
Mean SUV_max_ ± SD	8.0 ± 6.4 vs. 17.4 ± 11.9	8.0 ± 5.7 vs. 22.5 ± 12

Notes: CRS: Cytokine Release Syndrome; ICANS: Immune Effector Cell-associated Neurotoxicity syndrome; SUV_max_: maximum Summarized Uptake Value; vs.: versus. * indicates statistical significance.

**Table 4:** Overview of correlations of clinical parameters and SUV_max_.

Baseline Clinical Parameters and Adverse Events	Pre-Therapeutic SUV_max_		
	r	*p*	Odds: SUV_max_ > 17
LDH	0.5 CI: 0.04, 0.74	0.02 *	__
IL-6	0.1 CI: −0.46, 0.48	>0.05	__
CRP	0.2 CI: −0.29, 0.61	>0.05	__
mEASIX score	0.3 CI: −0.19, 0.67	>0.05	__
CRS	0.5 CI: 0.04, 0.78	0.03 *	CRS > 1: *p* = 0.03 OR = 22 CI 2, 314 *
ICANS	0.6 CI: 0.19, 0.83	0.01 *	ICANS > 1: *p* = 0.04 OR = 18 CI 1, 271 *

Notes: r: correlation coefficient; *p*: *p*-value; CI: 95% confidence interval; OR: odds ratio; CI: confidence interval; mEASIX: modified Endothelial Activation and Stress Index. CRS: Cytokine Release Syndrome; ICANS: Immune Effector Cell-associated Neurotoxicity Syndrome; LDH: lactate dehydrogenase; IL-6: interleukin-6; * indicates statistical significance.

**Table 5:** Correlations of baseline liver and spleen SUV_max_ with grade of CRS/ICANS.

	CRS		ICANS	
	r	*p*	r	*p*
SUV_max_ spleen	0.0 CI: −0.47, 0.47	0.91	−0.2 CI: −0.62, 0.30	0.48
SUV_max_ liver	0.1 CI: −0.38, 0.54	0.64	0.1 CI: −0.38, 0.54	0.75

Notes: r: correlation coefficient; *p*: *p*-value; CRS: Cytokine Release Syndrome; ICANS: Immune Effector Cell-associated Neurotoxicity Syndrome; CI: 95% confidence interval.

## Data Availability

Data cannot be shared publicly because of data protection regulations in Germany and the requirements of the ethics committee. Data are available from the institutional research committee of Duesseldorf Heinrich-Heine University (contact via study number 2023-2618) for researchers who meet the criteria for access to confidential data.
